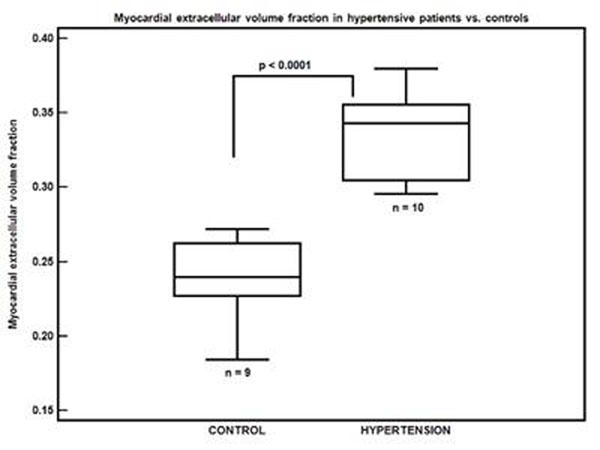# Identification of myocardial extracellular matrix expansion by cardiac MRI in hypertensive patients

**DOI:** 10.1186/1532-429X-13-S1-O109

**Published:** 2011-02-02

**Authors:** Francois-Pierre Mongeon, Michael Jerosch-Herold, Otavio Rizzi Coelho-Filho, Luciana F Seabra, Eri Watanabe, Ron Blankstein, Raymond Y Kwong

**Affiliations:** 1 Brigham and Women's Hospital, Boston, MA, USA

## Background

Left ventricular hypertrophy (LVH) is an important marker of adverse prognosis in cardiovascular disease and commonly occurs in association with hypertension. While late gadolinium enhancement (LGE) reflects myocardial replacement fibrosis in hypertensive patients, we hypothesized that the myocardial extracellular volume fraction (MECVF) would better reflect the presence and severity of myocardial fibrosis, including diffuse fibrosis, in hypertensive patients with LVH. We sought to measure the MECVF directly using T1 imaging pre- and post-contrast.

## Methods

We performed 3T cardiac MRI in 19 subjects including 9 normo-tensive control subjects (mean age 45±11 years, 66.7% female) and 10 patients (mean age 65±15 years, 40% female) with (1) systemic hypertension, (2) LVH by ECG or echocardiography, (3) a LVEF ≥ 50% and (4) no LGE. MRI included cine imaging and LGE 10 minutes after 0.15 mmol/Kg of gadolinium. In addition, a Look-Locker gradient echo technique with adiabatic inversion was used, once before and 3 times over a 30-min period after gadolinium injection, to quantify T1. The myocardial partition coefficient was estimated by least-squares linear regression of R1 (1/T1) in myocardium against R1 in blood. MECVF was obtained by adjusting the partition coefficient by the patient’s hematocrit.

## Results

The mean blood pressure (BP) at the time of MRI was not significantly different in treated hypertensive patients compared with control subjects (mean systolic BP 128 vs. 118 mmHg, p=0.1165; mean diastolic BP 69 vs. 74 mmHg, p=0.2930). Despite the presence of LVH by ECG or echocardiography, the median LV mass index by cardiac MRI only demonstrated a trend increase (p=0.0653) in hypertensive patients (53.8 g/m^2^ IQR 41.3-59.2) compared to controls (44.5 g/m^2^ IQR 36.5-48.3). However, there was evidence of increased MECVF in hypertensive patients (mean 0.34±0.03 vs. 0.24±0.03 in controls, p<0.0001, figure). There was no correlation between MECVF and LV mass index (r=0.32, p=0.17), and between MECVF and the E/E’ ratio by tissue Doppler echocardiography in hypertensive patients (n=8, r=0.24 p=0.53 for the lateral wall, r=0.12 p=0.75 for the septal wall).

## Conclusions

Patients with chronic hypertension show evidence of extracellular matrix expansion. We postulate that our method of direct quantification of extracellular matrix expansion by MECVF may better reflect the impact of increased afterload on the LV myocardium than LV mass or echocardiographic assessment of diastolic function. This novel quantitative method also holds promises for monitoring the effects of antihypertensive medication.

**Figure 1:**